# Supplementary material for: Gold Nanoparticle Mesoporous Carbon Composite as Catalyst for Hydrogen Evolution Reaction
Source: Molecules. 2024 Aug 5;29(15):3707. doi: 10.3390/molecules29153707 (PMC11314187; doi:10.3390/molecules29153707)
Supplement: Supplementary file 1 [file molecules-29-03707-s001.zip › molecules-3051120-supplementary.pdf]

Supplementary Materials

Gold Nanoparticle Mesoporous Carbon Composite as Catalyst for Hydrogen Evolution Reaction

Erik Biehler, Qui Quach and Tarek M. Abdel-Fattah *

Applied Research Center at Thomas Jefferson National Accelerator Facility, Department of Molecular Biology and Chemistry at Christopher Newport University, Newport News, VA 23606, USA

* Correspondence: fattah@cnu.edu

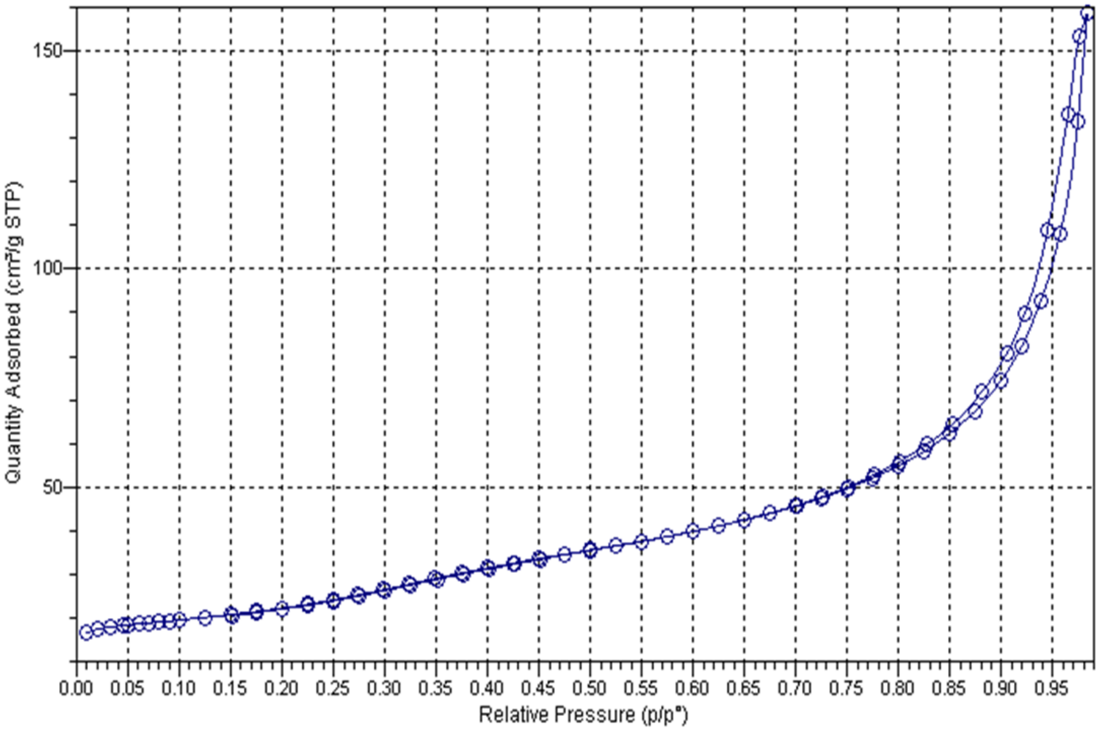

Figure S1. Nitrogen sorption/desorption isotherms generated at 77 K for Mesoporous Carbon.

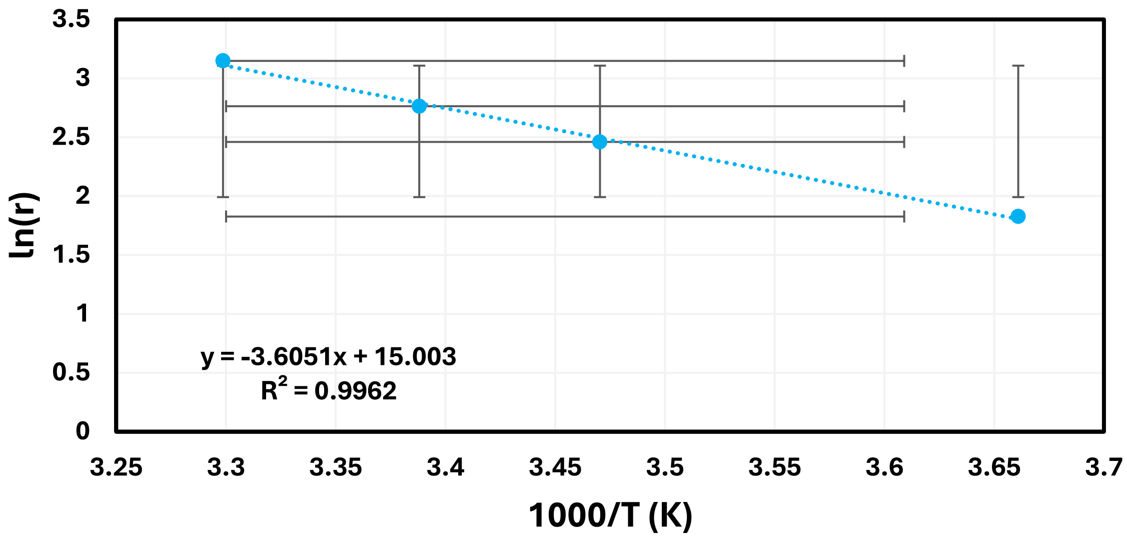

Figure S2. Arrhenius plot showing the Standard Deviation values for calculating the activation energy of a hydrogen generation reaction with the AuNP-MCM catalyst.

Table S1. Nitrogen Adsorption data for Mesoporous Carbon

**BET Surface Area (m^2/g)**	**79.7**
**Single point adsorption total pore volume of pores (cm^3/g)**	**0.246**
**BJH Adsorption average pore width (Å)**	**110**
